# Supplementary material for: Yiai Fuzheng decoction inhibits triple-negative breast cancer by remodeling the immune microenvironment
Source: Front Immunol. 2025 Sep 30;16:1615631. doi: 10.3389/fimmu.2025.1615631 (PMC12518410; doi:10.3389/fimmu.2025.1615631)
Supplement: Supplementary file 2 [file DataSheet1.zip › Supplementary File 2/Treatment/C_vs_H/07_Pathway_Analysis/Pathway_Analysis/Sig_Pathways_Name/Arachidonic acid metabolism.pdf]

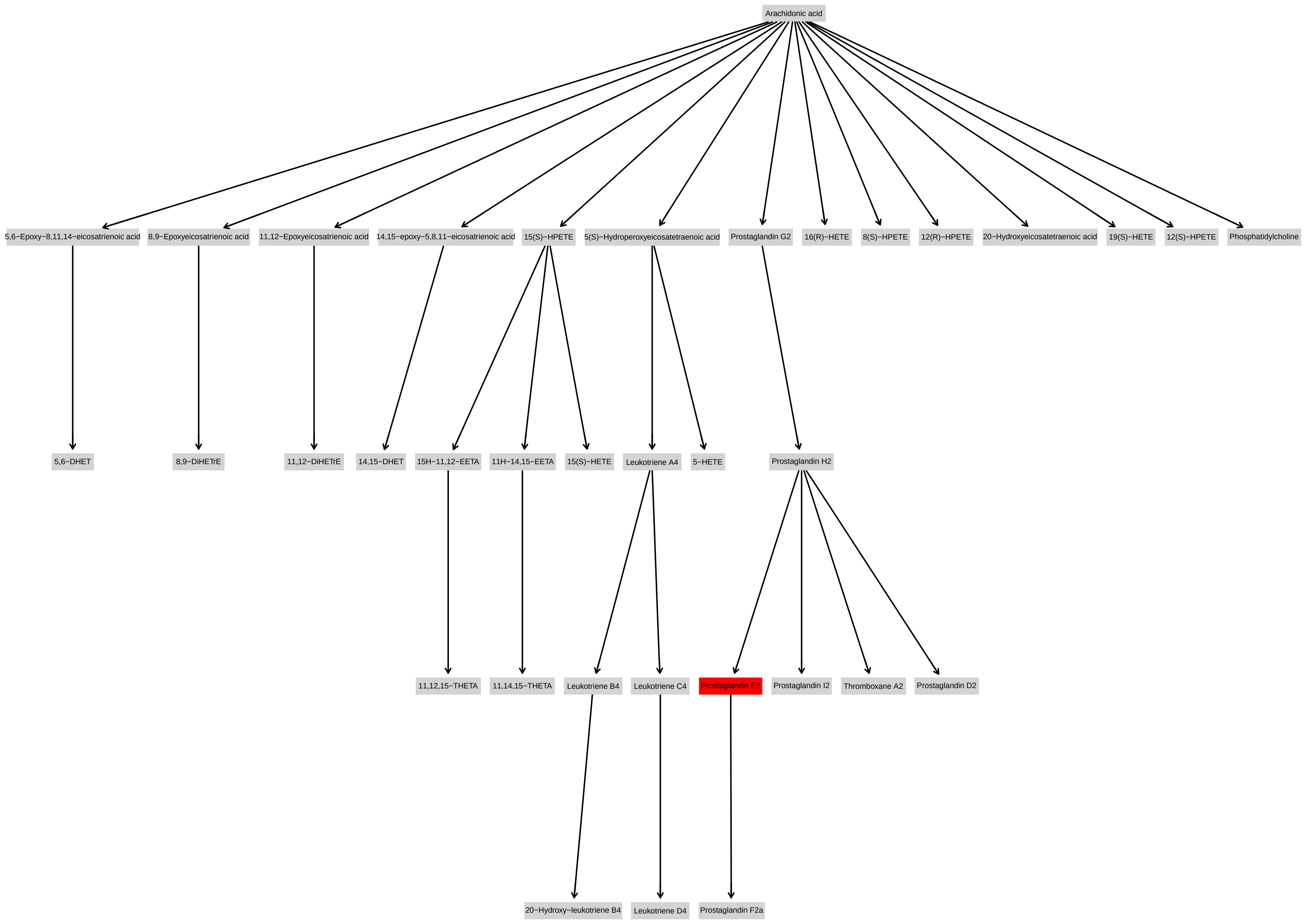
Arachidonic acid
5,6−Epoxy−8,11,14−eicosatrienoic acid
8,9−Epoxyeicosatrienoic acid
11,12−Epoxyeicosatrienoic acid
14,15−epoxy−5,8,11−eicosatrienoic acid
15(S)−HPETE
5(S)−Hydroperoxyeicosatetraenoic acid
Prostaglandin G2
16(R)−HETE
8(S)−HPETE
12(R)−HPETE
20−Hydroxyeicosatetraenoic acid
19(S)−HETE
12(S)−HPETE
Phosphatidylcholine
5,6−DHET
8,9−DiHETrE
11,12−DiHETrE
14,15−DHET
15H−11,12−EETA
11H−14,15−EETA
15(S)−HETE
Leukotriene A4
5−HETE
Prostaglandin H2
11,12,15−THETA
11,14,15−THETA
Leukotriene B4
Leukotriene C4
Prostaglandin E2
Prostaglandin I2
Thromboxane A2
Prostaglandin D2
20−Hydroxy−leukotriene B4
Leukotriene D4
Prostaglandin F2a
